# Effect and safety of tislelizumab combined with anlotinib and radiotherapy in advanced hepatocellular carcinoma: A single-arm, single-center, phase II clinical study

**DOI:** 10.1038/s41598-026-55609-3

**Published:** 2026-05-28

**Authors:** Huaxi Fan, Yuhong Liu, Guishu Wu, Mingyue Rao, Jing Zhang, Peipei Zhu, Jianwen Zhang

**Affiliations:** 1 Department of Oncology, Affiliated Hospital of Southwest Medical University, Luzhou, 646000 Sichuan People’s Republic of China; 2 Department of Oncology, The First People’s Hospital of Liangshan Yi Autonomous Prefecture, Liangshan, 615000 Sichuan People’s Republic of China; 3 Department of Gastroenterology, Dazhou Integrated Traditional Chinese and Western Medical Hospital, Dazhou, 635000 Sichuan People’s Republic of China; 4 Nuclear Medicine and Molecular Imaging Key Laboratory of Sichuan Province, Luzhou, 646000 Sichuan People’s Republic of China; 5 Academician (Expert) Workstation of Sichuan Province. . , Luzhou, 646000 Sichuan People’s Republic of China

**Keywords:** Hepatocellular carcinoma, Immune checkpoint inhibitor, Targeted therapy, Radiotherapy, Survival, Cancer, Oncology

## Abstract

Immune checkpoint inhibitors plus targeted therapy remains the mainstay of treatment for advanced hepatocellular carcinoma (HCC), yet its efficacy is limited. This study aimed to evaluate the efficacy and safety of tislelizumab combined with anlotinib and radiotherapy as first-line therapy for advanced HCC. This was a single-arm, single-center, phase II clinical trial. A total of 45 patients with advanced HCC were enrolled and administered first-line therapy consisting of tislelizumab plus anlotinib combined with radiotherapy. Stratification was performed according to pretreatment portal vein tumor thrombus (PVTT) status, Barcelona Clinic Liver Cancer (BCLC) stage, and median biologically effective dose (BED) of the gross tumor volume (GTV). Treatment response was assessed using the modified Response Evaluation Criteria in Solid Tumors, and adverse events (AEs) were graded per the National Cancer Institute Common Terminology Criteria for Adverse Events, version 4.0. The primary endpoint was the objective response rate (ORR). Secondary endpoints included the progression-free survival (PFS), overall survival (OS), disease control rate (DCR), and safety profiles reflected by AEs. The median BED of the GTV was 47.9 Gy (Gy). The ORR and DCR were 62.2% (28/45) and 75.6% (34/45), respectively. Median PFS and OS were 11.0 months (95% CI 1.444–14.081) and 26.7 months (95% CI 1.749–25.178), respectively. ORR and DCR were comparable across subgroups defined by PVTT status, BCLC stage, and BED level (all *p* > 0.05). No significant differences in PFS or OS were observed among these subgroups (all *p* > 0.05). Most AEs (82.2%, 37/45) were grade 1/2 and well tolerated. Grade 3/4 AEs occurred in 13.3% (6/45) of patients. No grade 5 events, radiation-induced liver disease, or treatment-related deaths were reported. Tislelizumab combined with anlotinib and radiotherapy demonstrates promising efficacy and acceptable safety as first-line treatment for advanced HCC. Clinical outcomes appear to be independent of PVTT status, BCLC stage, or radiation BED level.

Registration Number in the Chinese Clinical Trial Registry: ChiCTR2000039022 (10/13/2020). https://www.chictr.org.cn/index.html.

## Introduction

 Primary liver cancer (PLC) is the fourth most prevalent malignancy and the second leading cause of cancer-related mortality in China. Approximately 75–85% of PLC cases are pathologically diagnosed as hepatocellular carcinoma (HCC)^1^. The annual number of newly diagnosed HCC cases has increased by 55.0% since 2020, and the incidence is projected to rise by an additional 56.4% by 2040 compared with the 2020 baseline, reaching 1.4 million new confirmed cases^2^. Due to the insidious onset and atypical early clinical manifestations of HCC, 68.6% of patients are diagnosed at Barcelona Clinic Liver Cancer (BCLC) stage C, whereas only 12.8% present at stage B^3^. At present, therapeutic options for advanced HCC remain limited, and the clinical efficacy of single-modality treatment regimens remains unsatisfactory.

The advent of immune checkpoint inhibitors (ICIs) has brought new therapeutic hope for patients with advanced HCC. In the systemic treatment of advanced HCC, first-line monotherapy with targeted agents such as sorafenib and lenvatinib still yields limited clinical efficacy^4^. For ICI–targeted combination regimens, the median progression-free survival (PFS) ranges from 3.8 to 6.8 months^5–7^, and the median overall survival (OS) is approximately 16.4 months^7^. Although such immune-targeted combination strategies can prolong survival in advanced HCC, both PFS and OS benefits remain relatively modest, leaving substantial unmet clinical needs.

As an inflammation-associated malignancy (e.g., related to hepatitis B virus (HBV) and/or hepatitis C virus (HCV) infection)^8^, the progression and recurrence of HCC are closely associated with the tumor microenvironment (TME). A study by Gao et al. demonstrated that 33% of HCC TMEs are of the immune desert type, whereas only 17% exhibit a TME phenotype favorable for immunotherapy response^9^. Additionally, a study by Fu et al. indicated that the immunosuppressive nature of the TME promotes immune tolerance and tumor immune escape, thereby impairing the efficacy of immunotherapeutic interventions^10^. Therefore, triggering a more robust and specific anti-tumor immune response by remodeling the immunosuppressive TME remains a key challenge in HCC immunotherapy.

Anlotinib is a novel oral multi-target tyrosine kinase inhibitor. It suppresses multiple signaling targets, including vascular endothelial growth factor receptor and platelet-derived growth factor receptor, thereby exerting dual anti-angiogenic and anti-proliferative effects on tumor cells^11^. Tislelizumab is a humanized IgG4 monoclonal antibody targeting programmed death-1 (PD-1). By specifically binding to PD-1 expressed on the surface of T cells, it reverses tumor-mediated immunosuppression and restores and enhances T cell-driven anti-tumor immune responses^12^. As a classic local therapeutic modality, radiotherapy eliminates tumor cells by directly inducing DNA damage. Meanwhile, it synergizes with immunotherapy by triggering immunogenic cell death, increasing the release and exposure of tumor-associated antigens, and improving immune cell infiltration within TME^13–16^.

Based on the aforementioned mechanisms of action of each treatment modality and existing clinical evidence, the combination of immune-targeted therapy and radiotherapy is hypothesized to improve the objective response rate and prolong survival in patients with advanced HCC. Therefore, the present study aimed to evaluate the efficacy and safety of tislelizumab combined with anlotinib and radiotherapy as first-line treatment for advanced HCC, thereby providing a more effective therapeutic option for this patient population.

## Methods

### Study design

This was a single-arm, single-center phase II clinical trial registered with the Chinese Clinical Trial Registry (ChiCTR) on October 13, 2020 (Registration No.: ChiCTR2000039022; available at https://www.chictr.org.cn/index.html). The study protocol was approved by the Ethics Committee and Institutional Review Board of the Affiliated Hospital of Southwest Medical University on September 8, 2020 (Approval No.: KY2020135).

The primary objective was to evaluate the efficacy and safety of tislelizumab combined with anlotinib and radiotherapy as first-line therapy for advanced HCC, defined as unresectable BCLC stage B and C disease. Patients with advanced HCC who received this triple combination regimen as first-line treatment in the Department of Oncology, Affiliated Hospital of Southwest Medical University, between October 13, 2020, and December 31, 2023, were consecutively enrolled.

Efficacy assessments were performed by an independent review committee according to the modified Response Evaluation Criteria in Solid Tumors (mRECIST) version 1.1. Safety was evaluated using the National Cancer Institute Common Terminology Criteria for Adverse Events (NCI-CTCAE) version 4.0. Radiation-induced liver injury was graded per the Radiation Therapy Oncology Group/European Organization for Research and Treatment of Cancer (RTOG/EORTC) criteria. The primary endpoint was the objective response rate (ORR); secondary endpoints included OS, PFS, and disease control rate (DCR), and safety profiles reflected by adverse events (AEs).

**Fig. 1:**
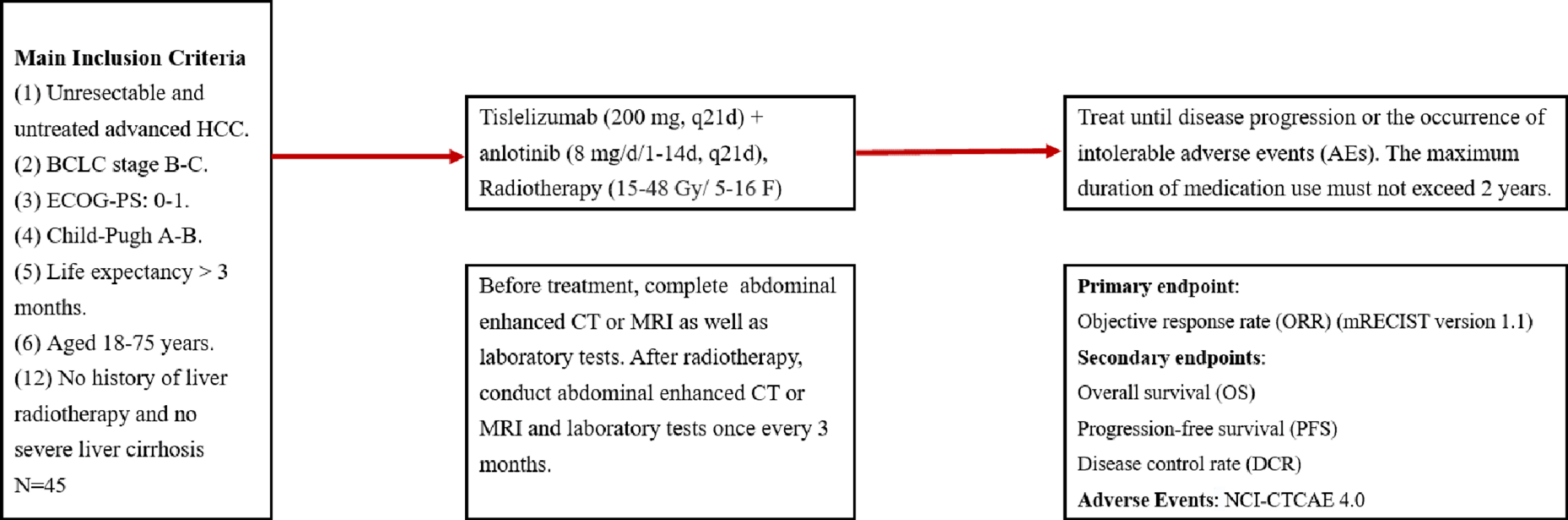
Study design. HCC, hepatocellular carcinoma; BCLC, Barcelona Clinic Liver Cancer staging system; ECOG, Eastern Cooperative Oncology Group; PS, performance status; CT, computed tomography; MRI, magnetic resonance imaging; mRECIST, modified Response Evaluation Criteria in Solid Tumors; NCI-CTCAE, National Cancer Institute Common Terminology Criteria for Adverse Events.

This study was conducted in accordance with the ethical principles of the 1975 Declaration of Helsinki and the Strengthening the Reporting of Observational Studies in Epidemiology (STROBE) statement. All patients provided written informed consent prior to enrollment and were free to withdraw from the study at any time. Radiation doses to the gross tumor volume (GTV) and clinical target volume (CTV) were reported as the biologically effective dose (BED) and equivalent dose in 2-gray (Gy) fractions (EQD2), using an α/β ratio of 10 for HCC. The study design is illustrated in Fig. 1.

### Participants

HCC diagnosis was established based on typical imaging findings (contrast-enhanced computed tomography [CT] or magnetic resonance imaging [MRI]) and alpha-fetoprotein (AFP) levels, or histopathology when imaging and AFP criteria were inconclusive^17,18^.

Inclusion Criteria: (1) Unresectable, previously untreated advanced HCC; (2) At least one measurable lesion; (3) Eastern Cooperative Oncology Group (ECOG) performance status 0–1; (4) Child–Pugh class A or B; (5) Adequate organ function: platelets ≥ 60 × 10^5^/L, hemoglobin ≥ 85 g/L, international normalized ratio ≤ 2.3, albumin (ALB) ≥ 28 g/L, total bilirubin (TBIL) ≤ 30 mg/L, alanine transaminase (ALT) aspartate transaminase (AST) ≤ 5 × upper limit of normal (ULN), serum creatinine ≤ 1.5 × ULN; (6) Expected survival > 3 months; (7) Age 18–75 years; (8) No prior liver radiotherapy or severe cirrhosis; (9) No history of severe allergy; (10) Voluntary acceptance of radiotherapy and written informed consent.

Exclusion Criteria: (1) Antitumor therapy within 3 months before enrollment; (2) Autoimmune disease or untreated gastrointestinal lesions with bleeding risk; (3) High bleeding risk in other organ systems; (4) Pregnancy or lactation; (5) Brain metastasis, hepatic encephalopathy, hematological disorders, or cardiac insufficiency; (6) Severe pulmonary dysfunction (e.g., silicosis, pulmonary fibrosis); (7) Moderate to severe hypothyroidism; (8) Inability to tolerate treatment or comply with follow-up.

All patients received laboratory tests and imaging examinations prior to treatment initiation. Laboratory evaluations encompassed cardiopulmonary function, thyroid function, liver function, renal function, complete blood count, and coagulation function, along with measurements of HBV-DNA and AFP levels. Imaging assessments included contrast-enhanced CT and/or contrast-enhanced MRI.

In this study, the treatment regimen consisted of tislelizumab combined with anlotinib plus radiotherapy. Tislelizumab (200 mg, intravenous infusion, every 21 days; Beigene, China) was administered as previously reported^12^. Anlotinib (8 mg per tablet) was orally taken once daily on days 1 to 14, followed by a 1-week drug-free interval before the next cycle; the agent was produced by Chiatai Tianqing, China^11^. All patients commenced radiotherapy on the second or third day after tislelizumab infusion. The combination therapy of tislelizumab plus anlotinib was administered for a minimum of 4 cycles. Treatment was discontinued in cases of disease progression or intolerable adverse reactions. The maximum duration of this combined regimen was limited to 2 years.

The detailed procedures of radiotherapy positioning and scanning requirements have been fully described in our previous study^19^. All patients were immobilized with a thermoplastic body mask, and abdominal compression was applied to minimize liver movement while maintaining normal breathing. Notably, the positioning simulation scan for target volume delineation must cover both the arterial and venous phases, and target volume delineation should be performed based on the venous phase images.

Target volumes were delineated strictly in accordance with relevant clinical guidelines^20^. GTV encompassed the primary tumor lesion, metastatic lymph nodes, and tumor thrombus. CTV was generated by expanding 5 mm outward from the GTV, and the planning target volume (PTV) was further expanded by 5 mm based on the CTV. Organs at risk (OARs) included the normal liver parenchyma, entire stomach, small intestine, bilateral kidneys, as well as the spinal cord, heart and lungs within 5 cm outside the PTV.

Intensity-modulated radiotherapy was delivered. The prescribed radiation doses were 15.0–48.0 Gy for the GTV and 13.0–42.0 Gy for the CTV, administered in 5–16 fractions (tumor α/β ratio = 10). The corresponding BED values were 19.5–62.4 Gy for the GTV and 16.4–52.4 Gy for the CTV; the corresponding EQD2 values were 16.3–52.0 Gy and 13.7–43.7 Gy, respectively. All treatment plans satisfied the following dose constraints. For the GTV and CTV, the dose covering 95% of the target volume (D95) was required to be ≥ 14.2–46.0 Gy (BED: 18.2–59.2 Gy; EQD2: 15.2–49.3 Gy) and ≥ 12.5–40.0 Gy (BED: 15.6–50.0 Gy; EQD2: 13.0–41.7 Gy), respectively. The mean dose to the normal liver parenchyma was limited to < 8.0–20.0 Gy (BED: 12.3–30.7 Gy; EQD2: 7.4–18.4 Gy, α/β = 3), and the mean dose to bilateral normal kidneys was < 10.0–20.0 Gy (BED: 16.7–30.7 Gy; EQD2: 10.0–18.4 Gy, α/β = 3). The maximum dose to the stomach was constrained to < 6.0–20.0 Gy (BED: 8.4–30.7 Gy; EQD2: 5.0–18.4 Gy, α/β = 3). The maximum dose to the small intestine and spinal cord was limited to 10.0–18.0 Gy (BED: 16.7–24.8 Gy; EQD2: 10.0–14.8 Gy, α/β = 3).

Starting from the first dose of tislelizumab, contrast-enhanced CT and/or MRI examinations, together with organ function assessments, were performed every 3 months. Organ function evaluations included coagulation function, thyroid function, routine blood tests, as well as detection of HBV-DNA and AFP levels. All patients were followed up every 3 months via outpatient revisit or telephone interview. Stratified analysis was conducted based on pretreatment portal vein tumor thrombus (PVTT) status, BCLC stage, and the median BED of the GTV. Patients were divided into PVTT-positive and PVTT-negative subgroups, BCLC stage B and stage C subgroups, as well as subgroups with BED higher than the median and lower than the median.

Treatment efficacy was assessed according to the mRECIST version 1.1. The response definitions were as follows: complete response (CR), complete disappearance of all target lesions; partial response (PR), a reduction of over 30% in the sum of target lesion diameters; stable disease (SD), a decrease of less than 30% or an increase of less than 20% in the sum of target lesion diameters; progressive disease (PD), an increase of more than 20% in the sum of target lesion diameters. ORR was defined as the proportion of patients achieving CR plus PR, and DCR was defined as the proportion of patients achieving CR plus PR plus SD.

According to the NCI-CTCAE version 4.0, acute AEs were assessed every two weeks within the first month of treatment and every three months thereafter. Acute toxicity was defined as AEs occurring within three months after the initiation of radiotherapy combined with tislelizumab and anlotinib, while late toxicity referred to AEs emerging more than three months following treatment commencement. RILD was defined as the presence of benign ascites accompanied by transaminase levels greater than twice ULN, which occurred between two weeks and three months after radiotherapy^21^.

### Statistical analysis

We adopted a one-sided test at a significance level α of 0.05 and a statistical power (1 − β) of 0.9. The anticipated dropout rate was set at 10–15%, and the expected ORR in this study was assumed to be 49.0%. Taking the ORR of 26.8% reported for first-line treatment of unresectable HCC with camrelizumab plus rivoceranib^22^ as the reference, the required sample size was calculated using PASS 15.0 software. The theoretical sample size without accounting for dropout was 38 patients. After adjustment for a 10–15% dropout rate, the corrected sample size ranged from 43 to 45 patients.

Statistical analyses were performed using SPSS 29.0 software. Categorical variables were compared using the chi-square test or Fisher’s exact test. Continuous variables were analyzed via the independent samples t-test or nonparametric tests. The Kaplan–Meier method was used to plot PFS and OS curves, and the Log-rank (Mantel–Cox) test was adopted for survival comparisons. P-value less than 0.05 was considered statistically significant.

## Results

### Patients

Table 1 summarizes the baseline clinical characteristics of the enrolled patients. For HBV- DNA- positive patients, entecavir 0.5 mg once daily (Dongrui Pharmaceutical Co., Ltd., China) was administered to maintain the HBV-DNA level below 1 × 10^2^ IU/mL.

The first patient was enrolled on September 20, 2020. Between September 20, 2020 and December 31, 2023, a total of 108 patients with advanced PLC were treated in the Department of Oncology, the Affiliated Hospital of Southwest Medical University. Among them, 47 patients with advanced HCC received first-line treatment consisting of tislelizumab combined with anlotinib and radiotherapy. Two patients were excluded due to failure to complete the planned radiotherapy course, leaving 45 patients who ultimately met the inclusion criteria. Of these eligible patients, 22 with AFP < 400 ng/mL were confirmed by histopathological diagnosis.

The median patient age was 49.0 years (range, 30.0–72.0 years). The median GTV and normal liver volume (NLV) were 432.5 cm^3^ and 807.6 cm^3^, respectively, with a median GTV-to-NLV ratio of 53.6%. The median radiation dose, median BED, and median EQD2 of the GTV were 36.9 Gy, 47.9 Gy, and 39.9 Gy, respectively.

In terms of treatment cycles, 5 patients completed 4 cycles of sequential tislelizumab plus anlotinib, 15 patients completed 5–12 cycles, and 25 patients completed 13–16 cycles. The follow-up period commenced on September 20, 2020, with the follow-up cutoff date set as October 31, 2025. The median follow-up duration was 20.5 months (range 9.9–60.0 months).

**Table 1:** Clinical characteristics of patients with advanced HCC.

Characteristics	All patients (*n* = 45)
Age (< 50/≥ 50 years, %)	23 (51.1)/22 (48.9)
ECOG score (0/1, %)	30 (66.7)/15 (33.3)
Gender (male/female, %)	44 (97.8)/1 (2.2)
BCLC stage (B/C, %)	10 (22.2)/35 (77.8)
ALBI grade (1/2, %)	38 (84.4)/7 (15.6)
Child-Pugh score (A/B, %)	28 (62.2)/17 (37.8)
AFP (≥ 400 ng/mL/ < 400 ng/mL, %)	23 (51.1)/22 (48.9)
Drinking (yes/no, %)	19 (42.2)/26 (57.8)
HBV-DNA (positive/negative, %)	30 (66.7)/15 (33.3)
Liver cirrhosis (yes/no, %)	23 (51.1)/22 (48.9)
Portal hypertension (yes/no, %)	22 (48.9)/23 (51.1)
Intrahepatic metastasis (yes/no, %)	33 (73.3)/12 (26.7)
Lymphatic metastasis (yes/no, %)	25 (55.6)/20 (44.4)
Extrahepatic metastasis (yes/no, %)	10 (22.2)/35 (77.8)
Ascites (yes/no, %)	11 (24.4)/34 (75.6)
Portal vein tumor thrombus (yes/no, %)	19 (42.2)/26 (57.8)
Portal vein invaded (yes/no, %)	21 (46.7)/24 (53.3)
Median volume of GTV (cm^3^)	432.5 (12.4–3198.7)
Median NLV (cm^3^)	807.6 (446.9–1673.6)
Median dose of GTV (Gy)	36.9 (15.0–60.0)
Median BED of GTV (Gy)	47.9 (19.5–78.0)
Median EQD2 of GTV (Gy)	39.9 (16.3–62.0)
Ratio of GTV to NLV (%)	53.6 (432.5/807.6)

HCC, hepatocellular carcinoma; ECOG, Eastern Cooperative Oncology Group; BCLC, Barcelona Clinical Liver Cancer; ALBI, albumin-bilirubin; AFP, alpha fetoprotein; HBV-DNA, hepatitis B virus deoxyribonucleic acid; GTV, gross tumor volume; NLV, normal liver volume; Gy, gray; BED, biological effective dose; EQD2, equivalent dose in 2 Gy fractions.

### Efficacy

**Fig. 2:**
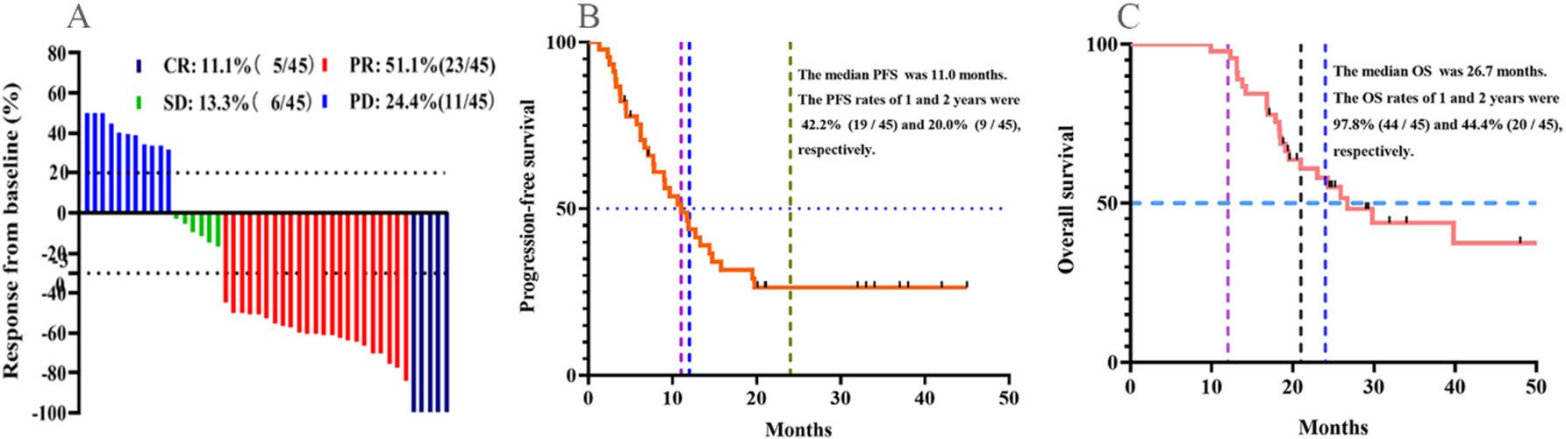
Treatment efficacy and survival outcomes in patients.

The CR rate and PR rate were 11.1% (5/45) and 51.1% (23/45), respectively (Fig. 2A). The corresponding ORR and DCR were 62.2% (28/45) and 75.6% (34/45). At the final follow-up, the median PFS was 11.0 months (95% CI 1.444–14.081) (Fig. 2B), and the median OS was 26.7 months (95% CI 1.749–25.178) (Fig. 2C). The 1-year and 2-year PFS rates were 42.2% (19/45) and 20.0% (9/45), while the 1-year and 2-year OS rates were 97.8% (44/45) and 44.4% (20/45), respectively. Among all enrolled patients, three who achieved PR were considered potentially eligible for subsequent surgical resection.

As shown in Fig. 2, the CR rate and PR rate were 11.1% (5/45) and 51.1% (23/45), respectively (Fig. 2A). The ORR and DCR were 62.2% (28/45) and 75.6% (34/45). The median PFS was 11.0 months (Fig. 2B), and the median OS was 26.7 months (Fig. 2C). The 1-year and 2-year PFS rates were 42.2% (19/45) and 20.0% (9/45), while the 1-year and 2-year OS rates were 97.8% (44/45) and 44.4% (20/45), respectively. CR, complete response; PR, partial response; SD, stable disease; PD, progressive disease; ORR, objective response rate; DCR, disease control rate; PFS, progression-free survival; OS, overall survival.

### Subgroup analysis

**Fig. 3:**
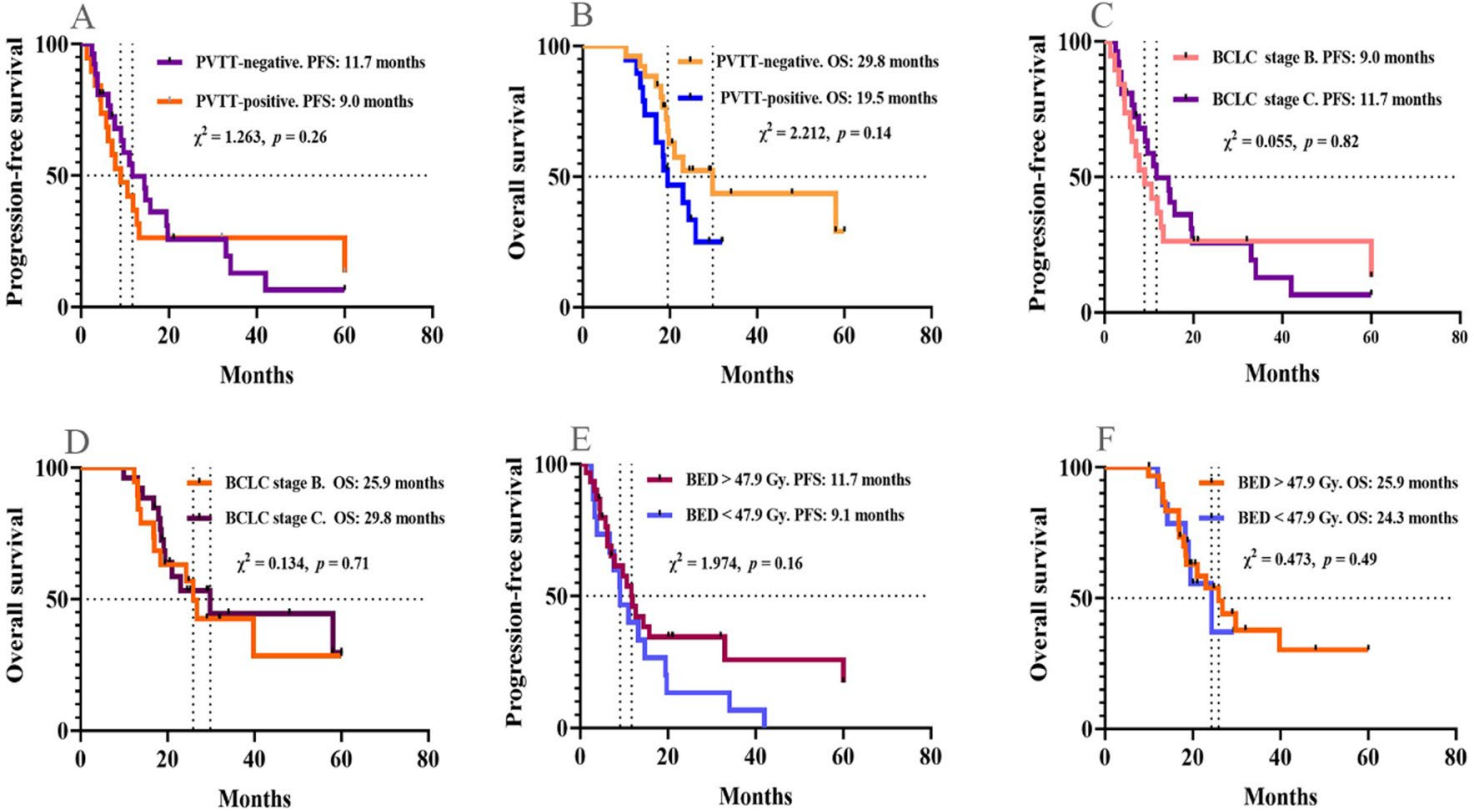
Survival analyses stratified by PVTT status, BCLC stage, and BED level.

The ORR and DCR were comparable between the PVTT-positive and PVTT-negative subgroups (68.4% vs. 57.7%, *p* = 0.54; 84.2% vs. 69.2%, *p* = 0.31, respectively) (Table 2). No significant differences in PFS and OS were observed between the two groups (11.7 vs. 9.0 months, χ^2^ = 1.263, *p* = 0.26, 95% CI 0.658–2.580, Fig. 3A and 29.8 vs. 19.5 months, χ^2^ = 2.212, *p* = 0.14, 95% CI 0.708–3.297, Fig. 3B, respectively).

Similarly, ORR and DCR were similar between patients with BCLC stage C and stage B (65.4% vs. 57.9%, *p* = 0.76; 80.8% vs. 68.4%, *p* = 0.49, respectively) (Table 2). No significant between-subgroup differences were found in PFS and OS (11.7 vs. 9.0 months, χ^2^ = 0.055, *p* = 0.82, 95% CI 0.393–1.499, Fig. 3C and 29.8 vs. 25.9 months, χ^2^ = 0.134, *p* = 0.71, 95% CI 0.389–1.939, Fig. 3D, respectively).

In addition, ORR and DCR were comparable between the BED > 47.9 Gy and BED < 47.9 Gy subgroups (60.0% vs. 66.7%, *p* = 0.75; 73.2% vs. 80.0%, *p* = 0.73, respectively) (Table 2). There were no significant differences in PFS and OS between the two BED subgroups (11.7 vs. 9.1 months, χ^2^ = 1.974, *p* = 0.16, 95% CI 0.660–2.518, Fig. 3E and 25.9 vs. 24.3 months, χ^2^ = 0.473, *p* = 0.49, 95% CI 0.185–1.076, Fig. 3F, respectively).

These results suggest that the PVTT-positive, BCLC stage C, and low-BED (< 47.9 Gy) subgroups achieved relatively higher DCR, while the PVTT-negative, BCLC stage C, and high-BED (> 47.9 Gy) subgroups had comparatively better survival outcomes.

**Table 2:** Comparisons of tumor responses among subgroups (*n* = 45).

Subgroups	CR (%)	PR (%)	SD (%)	ORR (%)	DCR (%)
PVTT-positive (*n* = 19)	2 (10.5)	11 (57.9)	3 (15.8)	13 (68.4)	16 (84.2)
PVTT-negative (*n* = 26)	3 (11.5)	12 (46.2)	3 (11.5)	15 (57.7)	18 (69.2)
BCLC stage B (*n* = 19)	3 (15.8)	8 (42.1)	2 (10.5)	11 (57.9)	13 (68.4)
BCLC stage C (*n* = 26)	2 (7.7)	15 (57.7)	4 (15.4)	17 (65.4)	21 (80.8)
BED > 47.9 Gy (*n* = 30)	3 (10.0)	15 (50.0)	4 (13.3)	18 (60.0)	22 (73.3)
BED < 47.9 Gy (*n* = 15)	2 (13.3)	8 (53.3)	2 (13.3)	10 (66.7)	12 (80.0)

PVTT, portal vein tumor thrombosis; BCLC, Barcelona Clinical Liver Cancer; BED, biological effective dose; CR, complete response; PR, partial response; SD, stable disease; ORR, objective response rate; DCR, disease control rate.

As shown in Fig. 3, no significant differences in PFS and OS were observed between subgroups: PVTT-positive versus PVTT-negative subgroups (11.7 vs. 9.0 months, χ^2^ = 1.263, *p* = 0.26, Fig. 3A and 29.8 vs. 19.5 months, χ^2^ = 2.212, *p* = 0.14, Fig. 3B); BCLC stage B versus stage C subgroups (9.0 vs. 11.7 months, χ^2^ = 0.055, *p* = 0.82, Fig. 3C and 25.9 vs. 29.8 months, χ^2^ = 0.134, *p* = 0.71, Fig. 3D); and BED > 47.9 Gy versus BED < 47.9 Gy subgroups (11.7 vs. 9.1 months, χ^2^ = 1.974, *p* = 0.16, Fig. 3E and 25.9 vs. 24.3 months, χ^2^ = 0.473, *p* = 0.49, Fig. 3F). PVTT, portal vein tumor thrombus; PFS, progression-free survival; OS, overall survival; BCLC, Barcelona Clinic Liver Cancer staging system; BED, biologically effective dose; Gy, Gray.

### Safety

Table 3 summarizes the incidence of AEs. Most treatment-related AEs were acute, with 82.2% (37/45) being grade 1/2 and clinically tolerable. AEs with an incidence rate exceeding 30.0% mainly included elevated transaminases, decreased albumin, fatigue, nausea/vomiting, and pruritus/rash. Six patients (13.3%, 6/45) developed grade 3/4 AEs. Transaminase and bilirubin levels gradually decreased, while ALB levels increased at 3–4 weeks after radiotherapy.

The incidence of grade 1/2 alanine transaminase elevation was significantly higher in the BED > 47.9 Gy group than in the BED < 47.9 Gy group (56.7% vs. 20.0%, *p* = 0.03). The incidence of grade 3/4 hypoalbuminemia was higher in the BED < 47.9 Gy group compared with the BED > 47.9 Gy group (20.0% vs. 0.0%, *p* = 0.03). No significant between-group differences were found in the incidences of other grade 1/2 and grade 3/4 AEs (*p* > 0.05). No grade 5 AEs, radiation-induced liver disease, or treatment-related fatal adverse events were observed.

**Table 3:** Adverse events graded per NCI-CTCAE version 4.0, n (%).

Adverse events	All patients (*n* = 45)		BED > 47.9 Gy (*n* = 30)		BED < 47.9 Gy (*n* = 15)		*p*
	Grade 1 / 2	Grade 3 / 4	Grade 1 / 2	Grade 3 / 4	Grade 1 / 2	Grade 3 / 4	
Hypothyroidism	19 (42.2)		13 (43.3)		6 (40.0)		> 0.05*
ALT	20 (44.4)	2 (4.4)	17 (56.7)		3 (20.0)	2 (13.3)	0.03*/> 0.05^#^
AST	19 (42.2)		14 (46.7)		5 (33.3)		> 0.05*
Hypoalbuminemia	17 (37.8)	3 (6.7)	12 (40.0)		5 (33.3)	3 (20.0)	> 0.05*/0.03^#^
Fatigue	16 (35.6)		13 (43.3)		3 (20.0)		> 0.05*
Nausea/vomiting	18 (40.0)		11 (36.7)		7 (46.7)		> 0.05*
Pruritus / Rash	14 (31.1)	1 (2.2)	9 (30.0)	1 (3.3)	5 (33.3)		> 0.05*^#^
Thrombocytopenia	13 (28.9)		9 (30.0)		4 (26.7)		> 0.05*
TBIL	11 (24.4)		10 (33.3)		1 (6.7)		> 0.05*
Leucopenia	12 (26.7)	2 (4.4)	10 (33.3)	1 (3.3)	2 (13.3)	1 (6.7)	> 0.05*^#^
Anemia	10 (22.2)	1 (2.2)	9 (30.0)		1 (6.7)	1 (6.7)	> 0.05*^#^
Electrolyte disturbance	6 (13.3)		5 (16.7)		1 (6.7)		> 0.05*
Diarrhea	5 (11.1)	1 (2.2)	5 (16.7)	1 (3.3)			> 0.05*^#^
Hypertension	5 (11.1)		4 (13.3)		1 (6.7)		> 0.05*
Constipation	3 (6.7)		3 (10.0)				> 0.05*
Pain	2 (4.4)		2 (6.7)				> 0.05*
Dyspepsia	2 (4.4)		1 (3.3)		1 (6.7)		> 0.05*
Myocarditis	1 (2.2)		1 (3.3)				> 0.05*

ALT, alanine transaminase; AST, aspartate aminotransferase; TBIL, total bilirubin; BED, biological effective dose; Gy, gray. *Comparisons of grade 1/2 adverse events between BED > 47.9 Gy and < 47.9 Gy groups. ^#^Comparisons of grade 3/4 adverse events between BED > 47.9 Gy and < 47.9 Gy groups.

## Discussion

Single-agent targeted therapy or ICIs yield limited therapeutic efficacy and survival benefits in patients with advanced HCC. As first-line treatment for advanced HCC, sorafenib achieves an ORR of 2.7–7.0% and a DCR of 28.7–55.0%, with a median PFS of 3.6 months and a median OS of 10.3–14.7 months^23,24^. For donafenib, the reported ORR and DCR are 4.6% and 30.8%, with a median PFS of 3.7 months and a median OS of 12.1 months^24^. For nivolumab, the ORR and DCR are 15.0% and 55.0%, respectively, with a median OS of 16.4 months^23^. Collectively, these data indicate that monotherapy with targeted agents or ICIs provides only modest efficacy and marginal survival improvement for advanced HCC. Therefore, exploring more effective combination treatment strategies is of great clinical significance.

Combination therapy with targeted agents and ICIs has become the mainstream first-line regimen for advanced HCC. The combination of camrelizumab plus rivoceranib yields an ORR of 26.8%, with a median PFS of 5.6 months and a median OS of 23.8 months^22^. The atezolizumab plus bevacizumab regimen achieves an ORR of 27.3% and a DCR of 73.6%, with a median PFS of 6.8 months; its 6-month and 12-month OS rates are 84.8% and 67.2%, respectively^5^. For sintilimab combined with a bevacizumab biosimilar, the ORR is 20.5% and the DCR is 72.0%, with a median PFS of 4.6 months^6^. The dual immune combination of nivolumab plus ipilimumab demonstrates an ORR of 36.0%, along with a median PFS of 9.1 months and a median OS of 23.7 months^25^. Collectively, these data confirm that combination regimens of targeted therapy and ICIs provide superior antitumor efficacy and survival benefits compared with single-agent targeted therapy or ICI monotherapy in advanced HCC. Nevertheless, the therapeutic outcomes and overall survival remain suboptimal and far from satisfying unmet clinical needs.

The results of this study demonstrated that the combination therapy of tislelizumab plus anlotinib and radiotherapy achieved significantly higher ORR and DCR, as well as prolonged median PFS and median OS, compared with conventional immune-targeted combination regimens for advanced HCC^5,6,22,25^. Additionally, the 1-year OS rate of the present regimen was superior to that of previously reported immune-targeted regimens^5^. These findings indicate that patients with advanced HCC may obtain substantial survival benefits from the combination of tislelizumab, anlotinib, and radiotherapy. Notably, this study was a single-arm clinical trial without a parallel control group. Therefore, indirect comparisons with published immune-targeted therapeutic regimens are inevitably associated with inherent limitations.

The results of stratified analysis based on PVTT status, BCLC stage, and BED level showed no significant differences in ORR, DCR, PFS, and OS among the respective subgroups. These findings suggest that the combination therapy of tislelizumab, anlotinib, and radiotherapy for advanced HCC may mitigate the adverse prognostic impacts of PVTT, advanced BCLC stage, and varying BED levels on tumor response and patient survival outcomes.

The potential mechanism underlying this favorable outcome may be attributed to the synergistic anti-tumor effects of the three treatment modalities: Tislelizumab restores the anti-tumor activity of T cells by blocking the programmed death-1 (PD-1)/programmed death-ligand 1 (PD-L1) signaling pathway, reversing immune escape of tumor cells; Anlotinib exerts dual anti-angiogenic and anti-tumor effects by inhibiting multiple targets, including vascular endothelial growth factor receptor and platelet-derived growth factor receptor, thereby improving the tumor microenvironment and enhancing the efficacy of immunotherapy; Radiotherapy directly eliminates tumor cells, releases tumor-associated antigens, and activates the body’s anti-tumor immune response, which in turn synergizes with the immunomodulatory effect of tislelizumab and the anti-angiogenic effect of anlotinib. The combination of these three modalities exerts anti-tumor effects through distinct but complementary mechanisms, ultimately improving the therapeutic effect and optimizing patient survival outcomes.

AEs of varying severity are commonly observed following single-agent targeted therapy, ICI monotherapy, and combined targeted-ICI regimens. For the nivolumab plus sorafenib regimen, the incidence of grade ≥ 3 AEs ranges from 1 to 14%^23^. Grade 3/4 AEs associated with camrelizumab plus rivoceranib mainly include hypertension (38%), palmar–plantar erythrodysesthesia syndrome (12%), and elevated transaminases (13%–17%)^22^. In the atezolizumab plus bevacizumab regimen, the incidence of grade 3/4 hypertension is 15.2%^5^. For sintilimab combined with a bevacizumab biosimilar, grade 3/4 AEs consist of hypertension (14%) and treatment-related death (2%)^6^. The overall incidence of grade 3/4 AEs in the nivolumab plus ipilimumab regimen reaches 41%, with fatal AEs accounting for 3.6%^25^.

In the present study, most AEs were acute reactions, predominantly grade 1/2 (82.2%) with good overall tolerability. The incidences of grade 1/2 AEs exceeding 30.0% were generally consistent with those reported in previous studies^5,6,22–24^. The incidence of grade 3/4 AEs in our cohort was 13.3%, which was lower than that observed in single-agent targeted therapy, single-agent ICI therapy, and conventional targeted–immune combination regimens.

BED-related AEs mainly manifested as grade 1/2 alanine transaminase elevation and grade 3/4 hypoalbuminemia, all of which improved after symptomatic treatment. No significant differences were found in the incidences of other AEs between subgroups. Importantly, no grade 5 AEs, radiation-induced liver disease, or treatment-related fatal adverse events were observed. Collectively, these findings indicate that the combination therapy of tislelizumab, anlotinib, and radiotherapy does not significantly increase the incidence of adverse events in patients with advanced HCC, and this triple combination regimen exhibits a manageable and favorable safety profile.

## Conclusion

Tislelizumab combined with anlotinib and radiotherapy showed good efficacy and safety in the treatment of advanced HCC, which demonstrated favorable ORR and DCR, prolonged PFS and OS, thus providing a new and effective treatment option for patients with advanced HCC. However, this study has some limitations: first, it is a single-arm, single-center, phase II clinical study with a relatively small sample size, which may lead to selection bias; second, the follow-up time is relatively short, so further extension of the follow-up period is needed for the evaluation of long-term efficacy and safety; third, further investigation was not performed to clarify the potential influence of biomarkers including programmed death-ligand 1 expression and genetic mutations, as well as the variability in systemic therapy cycles, on long-term survival outcomes; finally, no control group was set in this study, making it impossible to directly compare with other treatment regimens. In the future, multi-center, prospective, randomized controlled clinical trials are required to further verify the efficacy and safety of this combined treatment regimen.

## Data Availability

The original data related to the studies involved in this paper can be obtained by contacting the corresponding authors.
